# System evaluation of automated production and inhalation of ^15^O-labeled gaseous radiopharmaceuticals for the rapid ^15^O-oxygen PET examinations

**DOI:** 10.1186/s40658-018-0236-5

**Published:** 2018-12-19

**Authors:** Satoshi Iguchi, Tetsuaki Moriguchi, Makoto Yamazaki, Yuki Hori, Kazuhiro Koshino, Kazunori Toyoda, Jarmo Teuho, Saeka Shimochi, Yusuke Terakawa, Tetsuya Fukuda, Jun C. Takahashi, Jyoji Nakagawara, Shigehiko Kanaya, Hidehiro Iida

**Affiliations:** 1 Department of Radiology, National Cerebral and Cardiovascular Center, 5-7-1 Fujishiro-dai, Suita, Osaka 565-8565 Japan; 2 Graduated School of Information Science and Data Science Center, Nara Institute of Science and Technology, 8916-5 Takayama, Nara, 630-7192 Japan; 3 Department of Investigative Radiology, National Cerebral and Cardiovascular Center Research Institute, 5-7-1 Fujishiro-dai, Suita, Osaka 565-8565 Japan; 4 Department of Stroke and Cerebrovascular Diseases, National Cerebral and Cardiovascular Center, 5-7-1 Fujishiro-dai, Suita, Osaka 565-8565 Japan; 5 Turku PET Center, University of Turku and Turku University Hospital, Kiinamyllynkatu 4-8, 20520 Turku, Finland; 6 Department of Neurosurgery, National Cerebral and Cardiovascular Center, 5-7-1 Fujishiro-dai, Suita, Osaka 565-8565 Japan; 7 Integrative Cerebral and Cardiovascular Imaging Center, Department of Neurosurgery, National Cerebral and Cardiovascular Center, 5-7-1 Fujishiro-dai, Suita, Osaka 565-8565 Japan

**Keywords:** ^15^O-labeled oxygen (^15^O_2_), ^15^O-labeled carbon dioxide (C^15^O_2_), PET, Oxygen extraction fraction, Cerebral metabolic rate of oxygen

## Abstract

**Background:**

^15^O-oxygen inhalation PET is unique in its ability to provide fundamental information regarding cerebral hemodynamics and energy metabolism in man. However, the use of ^15^O-oxygen has been limited in a clinical environment largely attributed to logistical complexity, in relation to a long study period, and the need to produce and inhale three sets of radiopharmaceuticals. Despite the recent works that enabled shortening of the PET examination period, radiopharmaceutical production has still been a limiting factor. This study was aimed to evaluate a recently developed radiosynthesis/inhalation system that automatically supplies a series of ^15^O-labeled gaseous radiopharmaceuticals of C^15^O, ^15^O_2_, and C^15^O_2_ at short intervals.

**Methods:**

The system consists of a radiosynthesizer which produces C^15^O, ^15^O_2_, and C^15^O_2_; an inhalation controller; and an inhalation/scavenging unit. All three parts are controlled by a common sequencer, enabling automated production and inhalation at intervals less than 4.5 min. The gas inhalation/scavenging unit controls to sequentially supply of qualified radiopharmaceuticals at given radioactivity for given periods at given intervals. The unit also scavenges effectively the non-inhaled radioactive gases. Performance and reproducibility are evaluated.

**Results:**

Using an ^15^O-dedicated cyclotron with deuteron of 3.5 MeV at 40 μA, C^15^O, ^15^O_2_, and C^15^O_2_ were sequentially produced at a constant rate of 1400, 2400, and 2000 MBq/min, respectively. Each of radiopharmaceuticals were stably inhaled at < 4.5 min intervals with negligible contamination from the previous supply. The two-hole two-layered face mask with scavenging device minimized the gaseous radioactivity surrounding subject’s face, while maintaining the normocapnia during examination periods. Quantitative assessment of net administration doses could be assessed using a pair of radio-detectors at inlet and scavenging tubes, as 541 ± 149, 320 ± 103, 523 ± 137 MBq corresponding to 2-min supply of 2574 ± 255 MBq for C^15^O, and 1-min supply of 2220 ± 766 and 1763 ± 174 for ^15^O_2_ and C^15^O_2_, respectively.

**Conclusions:**

The present system allowed for automated production and inhalation of series of ^15^O-labeled radiopharmaceuticals as required in the rapid ^15^O-Oxygen PET protocol. The production and inhalation were reproducible and improved logistical complexity, and thus the use of ^15^O-oxygen might have become practically applicable in clinical environments.

## Background

^15^O-oxygen (^15^O_2_) PET is unique in its ability to provide fundamental information regarding cerebral hemodynamics and energy metabolism in man, which has proven central to understanding cerebral oxidative glycolysis in many pathologies, and it is considered the gold standard for identifying cerebral ischemia. Specifically, it has been utilized to quantitatively assess the regional cerebral metabolic rate of oxygen (CMRO_2_) and the regional oxygen extraction fraction (OEF) [1, 2]. In typical protocols, additional PET imaging had to be carried out in order to assess cerebral blood flow (CBF) using intravenous ^15^O-labeled water (H_2_^15^O) or inhalation of carbon dioxide (C^15^O_2_), and cerebral blood volume (CBV) using inhalation of ^15^O-carbon monoxide (C^15^O). The technique has contributed to the understanding of pathophysiology and to the development of guidelines for treating patients with stroke and cerebral vascular diseases [2]. More recently, a series of studies demonstrated that the aerobic glycolysis as characterized by means of a combined ^18^F-fluorodeoxyglucose (FDG) and ^15^O-oxygen PET imaging are related to malignancy in brain tumor [3], and also to potential alterations of the oxidative phosphorylation in patients with dementia [4] and more specifically with the amyloid deposition in Alzheimer disease [4–6].

Typical procedures in a PET examination with ^15^O-oxygen inhalation are labor intensive and have not been considered practical if one intends to utilize it in a clinical environment, largely attributed to the need for producing three different radiopharmaceuticals labeled with ^15^O in parallel to PET imaging [7]. Typical methods of the “three-step autoradiograph” [8] and the “steady-state method” [9–11] require an hour or even more for a full examination, during which the arterial blood sampling has often been needed with plasma separation to assess the radioactivity concentrations of ^15^O_2_ and H_2_^15^O in the arterial blood during the PET scan. Naive corrections are needed for radioactivity decay, and for the delay and dispersion of the arterial blood radioactivity curves [12, 13]. It should also be noted that quantitative imaging during inhalation of radioactive gases was challenging when using a three-dimensional (3D) PET scanner due to the increased random and scattered events originated from the gaseous radioactivity surrounding the patient face. Furthermore, internal radiation is also a legitimate concern for clinical staff unless a system is established to prevent unnecessary leakage of radioactive gases during patient inhalation while maintaining the normocapnia.

Recently, significant progress has been reported in the instrumentation and reconstruction software implemented in commercial 3D PET scanners. High-speed electronics offer precise data acquisition with considerably reduced random events. Consequently, the accuracy and signal-to-noise ratio are greatly improved [14], allowing theoretical model-based scatter compensation [15–20] to be accurately applied. A markedly improved performance in 3D PET is essential for better image quality and increased accuracy, while simultaneously reducing the radiation dose in patients [21]. It has also been demonstrated that the degradation in the quantitative accuracy has been resolved even during the inhalation of ^15^O-gases in 3D PET by means of the improvement of the scatter correction procedures [22], suggesting that the arterial input function (AIF) assessed with arterial blood sampling may be replaced by the image-derived AIF [23]. It should be noted that the kinetic model for ^15^O-oxygen has been well validated [24]. This also support the adequacy of a sophisticated kinetic formulation which shortens the PET examination period, by means of a 6–9 min single dynamic PET imaging while ^15^O_2_ and H_2_^15^O (or C^15^O_2_) are sequentially administered [25, 26]. Such a method has been well validated to provide quantitative OEF values that were consistent with those determined from the arterial-venous difference of oxygen contents in experimental animals (monkeys) for a wide physiological range [26].

The logistical complexity remains the major limitation in producing a series of ^15^O-labeled radiopharmaceuticals. The authors then hypothesized that this may be overcome by using an automated system that enables sequential production and inhalation of the ^15^O-labeled radiopharmaceuticals with minimal human operation and recording of all signals from the radiopharmaceutical production/inhalation system, and other peripheral equipment utilized in the ^15^O-Oxygen PET experiment. Recently, a system has been developed to produce and inhale a series of ^15^O-labeled radiopharmaceuticals of C^15^O, ^15^O_2_, and C^15^O_2_ sequentially with minimal human operations, as required in given protocols of the three-step autoradiography [8] and the rapid protocol of Kudomi et al. [25, 26]. The system for the radiopharmaceutical production and inhalation has been approved as a medical device in authors’ country, and contributed to clinical examinations on more than 2500 patients. The present study was then aimed to evaluate retrospectively the reproducibility of production and inhalation of radiopharmaceuticals, when applied to the ultra-rapid ^15^O-oxygen PET examinations. We then investigated the radioactivity recovery, and chemical and radiochemical purity in the ^15^O_2_ and C^15^O productions for different carrier gas concentrations. We further evaluated impact of reducing the leakage of radioactive gases from the inhalation face mask, and potential ability to estimate the administration dose to individual patients.

## Materials and methods

### Subjects

The automated radiosynthesis/inhalation system approved as a medical device in November 2012 in authors’ country has been utilized on more than 2500 clinical scans with ^15^O-oxygen inhalation following the protocol of Kudomi et al. [26]. Basic performance including the recovery, and chemical and radiochemical purity was evaluated for each of three gaseous radiopharmaceuticals of C^15^O, ^15^O_2_, and C^15^O_2_. Data previously conducted as clinical examinations between November 20, 2012 and March 31, 2015 (2.4 ± 1.2 examinations per day) were retrospectively analyzed. The data were from PET examinations on 749 patients, and included (a) the time chart log of the radiosynthesizer, which includes the time-radioactivity curves of the radio-detectors and gas chromatograms; (b) AIF recorded by the functional image calculation software; (c) record of Capnometer; (d) records of the time-radioactivity curves of the area monitor in a PET room, of the air in the exhaust duct from a PET area, and the cyclotron area; and (e) radioactivity curves of a pair of beta-ray detectors placed each of inlet and scavenge tubes of the facemask with the flow values for each tubes. The patients were 433 males and 316 females with a body weight of 60.4 ± 11.4 kg and an age of 59.8 ± 26.0 years old. The study was approved by the Ethics Committee at the authors’ institution with the approval number M30-013 for retrospective data analysis on clinical data and M22-089-2 for PET imaging on healthy volunteers.

### System description

**Fig. 1:**
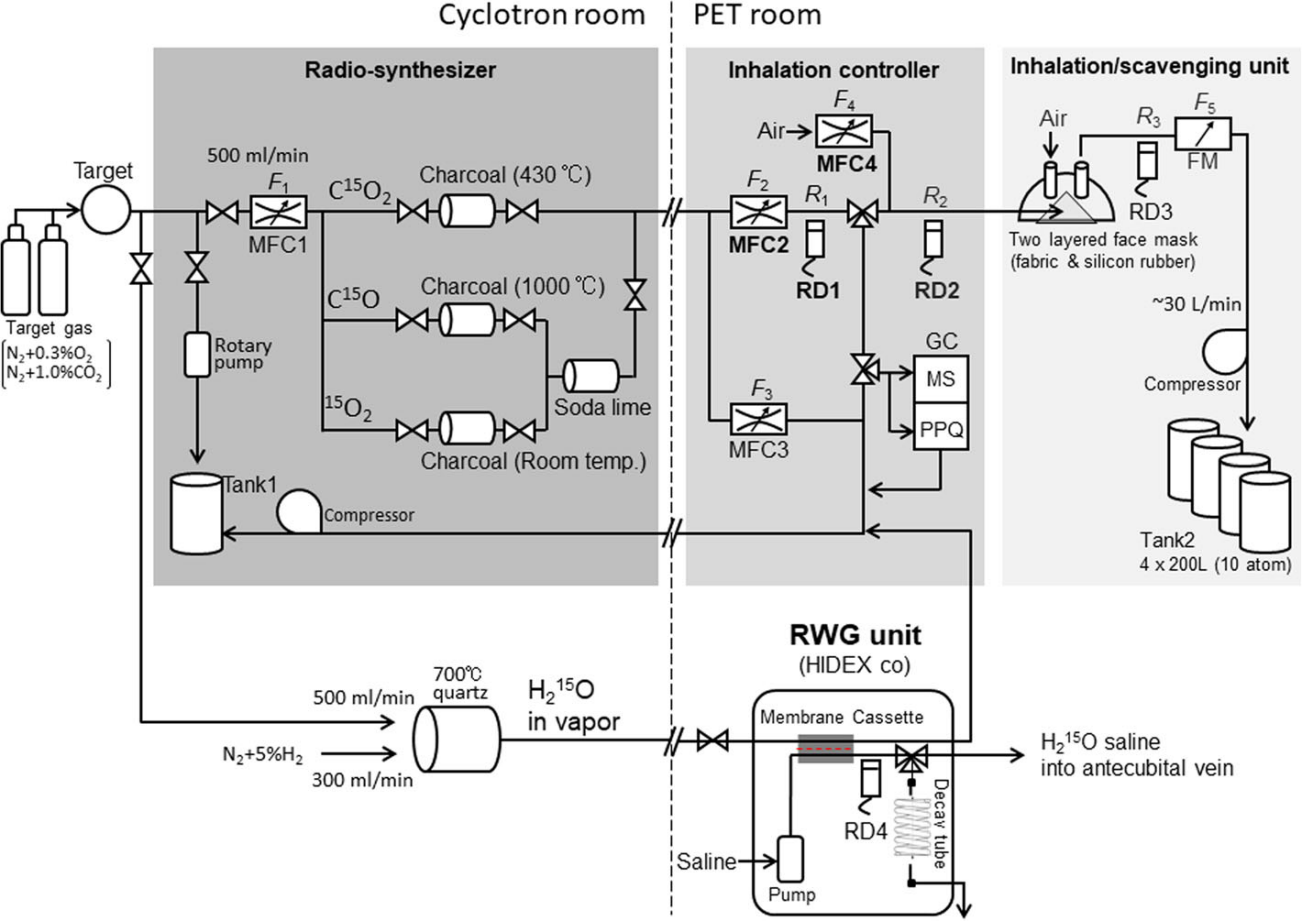
Schematic diagram of the system developed for ^15^O-oxygen PET, which consists of a radiosynthesizer installed in a cyclotron room, and an inhalation controller and an inhalation/scavenging unit installed in a PET room. An additional radio water generator (RWG) unit is installed in parallel to this system. MFC, RD, and FM indicate a mass flow controller, a radioactive detector, and a flow meter, respectively. *F* and *R* denote values for flow and radioactivity concentrations, respectively. All radio-detectors are calibrated to a standard dose calibrator (Model CRC712, CAPINTEC, Florham Park, NJ, USA). Two sets of gas chromatographs (GCs) are placed in the inhalation controller. See text

Figure 1 shows a diagram of the automated radiopharmaceutical production/inhalation system evaluated in this study. The system consists of three parts: (a) a radiosynthesizer, (b) an inhalation controller, and (c) an inhalation/scavenging unit. The system is combined with a cyclotron with a capability of accelerating a deuteron beam. In this study, a Cyclone 3D (C3D), dedicated to ^15^O production from IBA Radiopharma Solutions (Louvain-La-Neuve, Belgium), is used. The deuteron beam was approximately 3.5 MeV at a beam current of 40 μA on the target. An additional radio water generator (RWG) from HIDEX (Turku City, Finland) was placed to produce ^15^O-labeled water (H_2_^15^O) in parallel to the present system.

Each radioactive gas was continuously circulated during the beam bombardment to the target from the cyclotron target to the radiosynthesizer, the inhalation controller, and to a decay tank (tank 1, in Fig. 1). Radiopharmaceuticals were then supplied to the patient’s face mask from the inhalation controller for a specified period at the prescribed supply rate for each radiopharmaceutical in units of MBq/min, which can be adapted to a short-period inhalation protocol such as three-step autoradiography [8, 27] or a continuous supply as required in the steady-state method [11, 28]. The target chamber was actively evacuated at the end of each radiopharmaceutical supply to minimize the intervals between sequential inhalations of different radiopharmaceuticals. Due to avoidance contamination from the previous supply, the system can be applied to the multiple tracer approaches of Kudomi et al. [25, 26]. The production and supply of a series of ^15^O-gaseous radiopharmaceuticals were controlled by a sequencer, which allowed for automatic production. The system was also designed to accommodate changes in the order of sequential radiopharmaceutical productions, the amount of radioactivity, and the period of the inhalation. This study employed a fixed protocol in which where the radiosynthesis and in halation was first C^15^O followed by a sequential supply of ^15^O_2_ and C^15^O_2_ at a 4.5-min interval.

The system recorded all mechanics operations and count rates of the radioactivity detectors and flow values, which were transferred to the workstation to confirm the consistency with the PET data. Additional data were also recorded from other peripheral devices utilized during the PET examination, namely the arterial input function detector [29], a well counter, a Capnometer, and a pair of radioactivity detectors for inlet and scavenge tubes of the patient facemask. All these data were stored in a common workstation, and were used for quality assurance of the entire PET examination procedures.

Due to the requirement for radiation safety regulation in the authors’ country, air of the entire PET room was actively exhausted at a flow rate of approximately 2000 m^3^ per hour, which corresponded to 17–20 air changes per hour. The exhausted air from other four rooms was gathered into a single duct. Total air flow was approximately 7000 m^3^/h, as assessed according to the authorized procedures of JIS A1413 (equivalent to a proposed ISO 16640) using a hybrid flow meter (DP70, Hiyoshi-Denki, Yokohama City, Japan). A calibrated gamma-ray monitor (DGM-151, Hitachi-Aloka, Tokyo, Japan) was placed inside the exhaust duct to monitor the gaseous radioactivity carried from the PET room, and also from the entire facility during the PET examination period.

#### Radiosynthesizer

^15^O-radioactivity and the radiosynthesis of C^15^O, ^15^O_2_, and C^15^O_2_ followed the method described by Clark and Buckingham [7]. Briefly, ^15^O was produced by the ^14^N(d,n)^15^O nuclear reaction in the cyclotron target, in which the target gas to produce ^15^O_2_ was nitrogen (N_2_) containing oxygen (O_2_) as a carrier gas. The concentration of the carrier gas of O_2_ was chosen at 0.3% in all clinical studies according to a systematic evaluation in this study (see below). The ^15^O_2_ produced in the target was carried to a soda lime column to remove contamination, which was mostly C^15^O_2_. C^15^O_2_ was produced also in the target filled with N_2_ containing 1.0% of CO_2_. The C^15^O_2_ produced in the target was funneled to a charcoal column heated to 430 °C to remove contamination of C^15^O. C^15^O was synthesized from the ^15^O_2_ gas by carrying it away from the target into a charcoal column heated to 1000 °C. A soda lime column was then used to remove contamination, mostly C^15^O_2_. Each of the radioactive gases produced were continuously carried to the inhalation controller placed in the PET scanner room during deuteron beam bombardment and then to a compressing tank (tank 1 in Fig. 1). The flow rates to the radiosynthesizer from the target and to the inhalation controller were controlled by a mass flow controller (MFC) (MFC1 set at 500 mL/min).

Immediately after the end of each radiopharmaceutical production, the cyclotron beam was stopped. Then the target chamber was evacuated at a negative pressure using a rotary pump for a preset period of 18 s. The target chamber was then filled with a new target gas, and was exposed to the deuteron beam for the next radiopharmaceutical production.

#### Inhalation controller

While the synthesizer was producing radioactive radiopharmaceuticals, a radioactive gas was carried to the inhalation controller installed in the PET room and circulated to the waste tank (tank 1) in the cyclotron room. This gas was released from tank 1 after sufficient decay. The inhalation controller then supplied radioactive radiopharmaceuticals to a patient’s face mask according to the given protocols. In the clinical protocols at our institution, the supply flow rate defined as (*F*_2_ *+ F*_4_) was 1000 mL/min by controlling MFC2 and MFC4. The radioactivity supply rate, defined as *F*_2_ × *R*_1_, was at approximately 1300 MBq/min, 2400 MBq/min, and 2000 MB/min, respectively. The supply period for each radiopharmaceutical was 2 min, 1 min, and 1 min, yielding the total radioactivity supply, defined as (*F*_2_ *+ F*_4_) × *R*_2_ × the supply period, was to be 2600 MBq, 2400 MBq, and 2000 MBq corresponding to C^15^O, ^15^O_2_, and C^15^O_2_, respectively.

A gas chromatograph in the inhalation controller was aimed to confirm radiochemical and chemical purity, including contaminations of residual radiopharmaceuticals from the previous administration. A small gas sample was collected to assess the radiochemical and chemical purity using two small rapid gas chromatographs (CP-490, Agilent Technologies, Santa Clara, CA, USA), implemented Molecular Sieve 5A (MS) at 120 °C and Pora-Plot Q (PPQ) at 60 °C with capillary columns that are 10 m long, respectively. The gas samples were taken prior to each initiation of the radiopharmaceutical supply.

#### Inhalation/scavenging unit

**Fig. 2:**
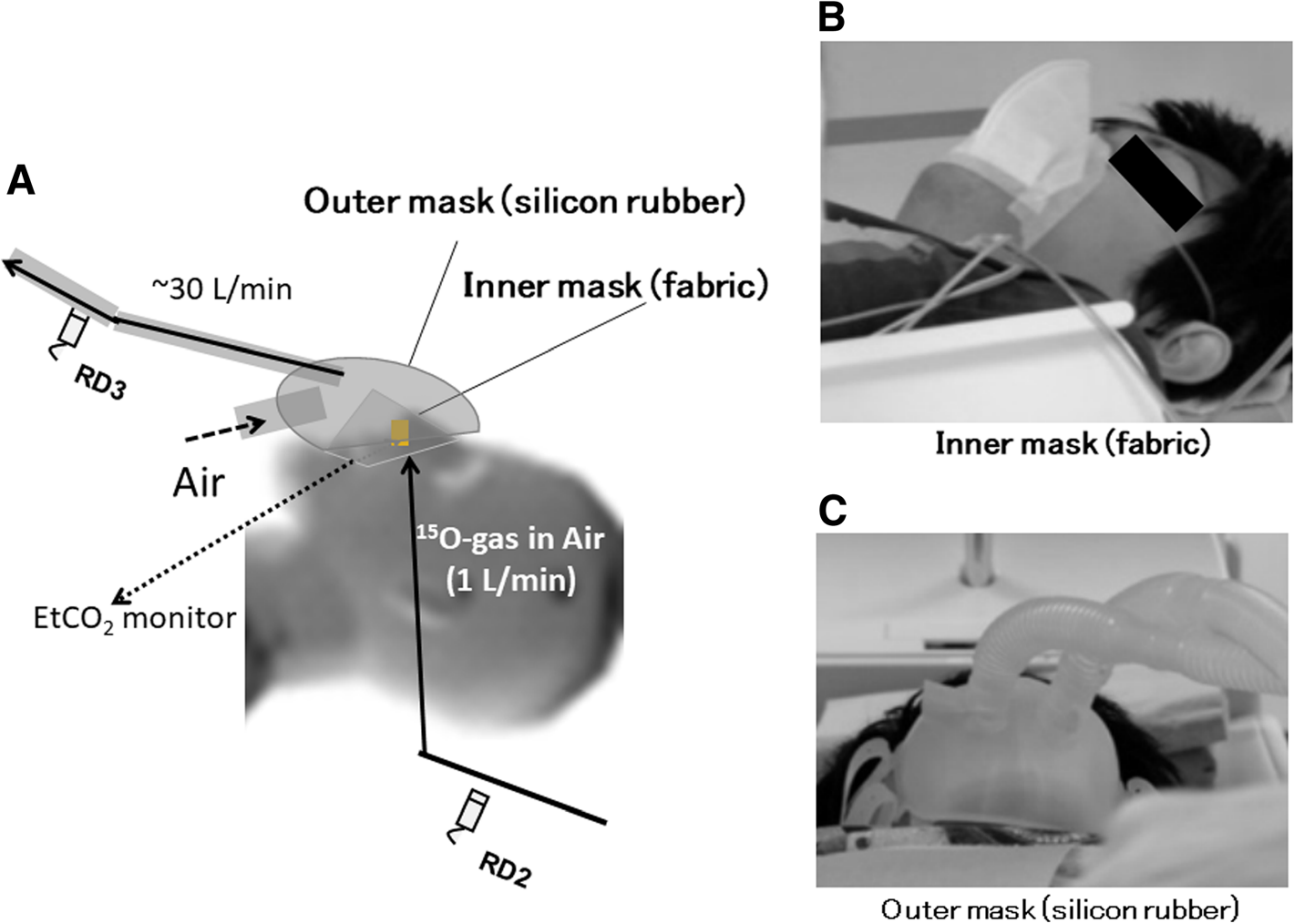
Two-hole two-layered face mask (**a**) implemented in the inhalation/scavenging unit of a ^15^O-oxygen production/inhalation system. Radioactive gases are supplied inside the inner mask made of fabric (**b**), and a patient breathes in the radioactive gases during the inhalation period. Leaked radioactivity from the inner face mask is scavenged at a flow rate of approximately 30  L/min, to tank 2 through the scavenging tube connected to one of the holes in the outer mask (**c**). Inlet tube for the outer face mask is open so that fresh air can be continuously supplied while the face mask is on the patient’s face

The inhalation/scavenging unit was designed to maintain the normocapnia of a patient during PET examination by introducing fresh air into the face mask while scavenging the gaseous radioactivity inside of the facemask to the decay tank (tank 2). The unit consisted of a two-hole two-layered face mask (Fig. 2a), a compressor system to scavenge radioactive gas from the face mask through one of the holes to the decay tanks, and a pair of radioactivity detector, RD2 and RD3 (Fig. 1). The flow rate in the scavenging tube was typically 30 L/min, as measured using a mass flow meter (FM in Fig. 1). The inner mask, which was made of fabric (Fig. 2b), confined gaseous radioactivity to a small area between the nose and the mouth. The outer face mask (Fig. 2c) was made of a 3.0-mm-thick silicon rubber to minimize leakage of radioactive air to the room. The thickness was defined to effectively stop the beta-rays emitted from gaseous ^15^O inside of the facemask, enabling accurate delineation of the face mask contour [22] and thus accurate correction of scatter by means of the single-scatter simulation method [18].

Scavenged air was compressed into tank 2, which consisted of four of 200-L tanks, where the storage was automatically changed among the tanks, each of which was released after sufficient decay. Capnometer (MSC8000, Contec Medical Systems, Hebei Provience, China) was used to monitor the end-tidal carbon dioxide partial pressure and the respiration rate during PET scanning. The tube for this Capnometer was also used to supply radioactive gases through a small-diameter tube into a space inside the inner layer of the facemask between the nose and the mouth.

#### Radio water generator unit

A radio water generator (RWG, HIDEX co, Turku, Finland) was placed in parallel to the ^15^O-oxygen production/inhalation system (see Fig. 1). ^15^O_2_ gas was continuously produced in the cyclotron target, and was mixed with a 5% H_2_ before the furnace heated at 700 °C to produce H_2_^15^O in the cyclotron room. The gas containing H_2_^15^O was then continuously circulated to a decay tank (tank 1) via the RWG unit installed in the PET room (Fig. 1). The radioactive H_2_^15^O was then transferred to the saline through a membrane cassette in the RWG device. The radioactive H_2_^15^O was continuously circulated to an outlet tube, from which a given amount of radioactive saline could be administered into the patient’s vein at the prescribed dose for a given period. A syringe sample of H_2_^15^O could also be taken from this tube, for an experimental use such as calibration, and quality assessment.

### Experimental procedures

All experiments and clinical scans were carried out by a deuteron beam bombardment of 40 μA on a target. The pressure of the target gas was 0.08 MPa. The flow rate of MFC1 in the synthesizer was 500 mL/min, and the supply flow rate to the face mask (MFC2 + MFC4) was 1000 mL/min.

The recovery and the dependency on the carrier gas concentration of O_2_ was evaluated for a production of gaseous ^15^O_2_, and consequently synthesized gaseous C^15^O and H_2_^15^O in saline. The experiment was carried out for a target gas of N_2_ containing 0.3%, 0.5%, and 1.0% carrier gas of oxygen (O_2_). ^15^O_2_ and C^15^O were continuously produced and carried to the inhalation unit. The radioactivity recovery was determined for a 1-min supply for both ^15^O_2_ or C^15^O. Gaseous ^15^O_2_ was also carried to RWG to generate H_2_^15^O saline, and the recovery was determined for 1-min sampling of H_2_^15^O in the saline. The radioactivity recovery of C^15^O_2_ was also assessed similarly using a target gas containing 1.0% carbon-dioxide (CO_2_) in N_2_. The radiochemical purity and non-radiochemical purity were assessed for each of C^15^O, ^15^O_2_, and C^15^O_2_ productions. These measurements were repeated six times for each radiopharmaceutical.

The radiochemical contamination was determined in the second administration namely C^15^O_2_ when the sequential ^15^O_2_–C^15^O_2_ inhalation protocol at 4.5-min interval was utilized as employed in the clinical examination. The radioactivity was assessed with and without producing the second radiopharmaceutical of C^15^O_2_. This experiment was also repeated six times.

### Retrospective analysis

Retrospective analysis was carried out using the data recorded for 749 subjects who underwent all C^15^O, ^15^O_2_, and C^15^O_2_ inhalation scans according to the fixed protocol, with inhalation periods of 2 min for C^15^O, and 1 min for ^15^O_2_ and C^15^O_2_. Radioactivity supply as calculated as an integral of the radioactivity concentration in the supply tube multiplied by its flow rate, and the net administration as calculated as an integral of the radioactivity concentration in the scavenging tube multiplied by its flow rate for each of C^15^O, ^15^O_2,_ and C^15^O_2_ inhalation scans. The net administration dose was then calculated as a subtraction of those two values for each radiopharmaceutical. Reproducibility of supply and net administration dose among subjects were evaluated. Time-dependent net administration radioactivity was also compared with the arterial input function (AIF) estimated from the continuously monitored arterial radioactivity time-activity curves (TACs).

The effective administration dose was divided by the body weight of the patient for the C^15^O_2_ inhalation scan and was compared with AUCs of the directly assessed AIF for each patient scan. It should be noted that the delay and dispersion were carefully compensated in AIFs as described in earlier works [12, 13, 30]. The integration period was set for the early period of 150 s after initiation of C^15^O_2_ inhalation.

Scavenging effectiveness of the two-hole two-layered facemask was evaluated on reconstructed tomographic and maximum intensity projection (MIP) images during ^15^O_2_ and C^15^O_2_ inhalation periods. Evaluation was also carried out on functional images of CBF, CMRO_2_, and OEF calculated according to two mathematical formulations, i.e., the DARG method [25] (Fig. 9a) and DBFM method [26] with a modification to estimate the arterial vascular components for both H_2_^15^O and ^15^O_2_, denoted as *V*_0_^W^ and *V*_0_^O^, respectively.

Leakage of the ^15^O-gaseous radioactivity to the PET room was calculated using the detector placed in the exhaust duct from the PET room, which was calibrated to the common dose calibrator utilized in our PET examination so that the detectors’ sensitivity was consistent with that of supply from the radiosynthesis/inhalation device. The known background level of 0.020 Bq/mL was subtracted from the observed counting rates of the detector in the exhaust duct. The total radioactivity from the PET room was then calculated by multiplying the flow rate of the duct. The radioactivity curves were then corrected for the radioactive decay of ^15^O at each time point back to the inhalation initiation time. The clearance of the gaseous radioactivity was interpolated by a single exponential function with a half-life of 3.5 min, which corresponded to 17 changes of the air in the PET room per hour, as estimated from the room volume and the flow rate. AUCs of the radioactivity concentration curves were calculated for a period of each radio-gas supply, and were then divided by the amount of radioactivity supply to each patient, so as to estimate the %leakage for each administration.

All data were presented as the mean ± 1 s.d. Pearson’s correlation analysis and linear regression analysis were used to evaluate the relationships between the two values. A value of *p* < 0.05 was considered statistically significant.

## Results

**Fig. 3:**
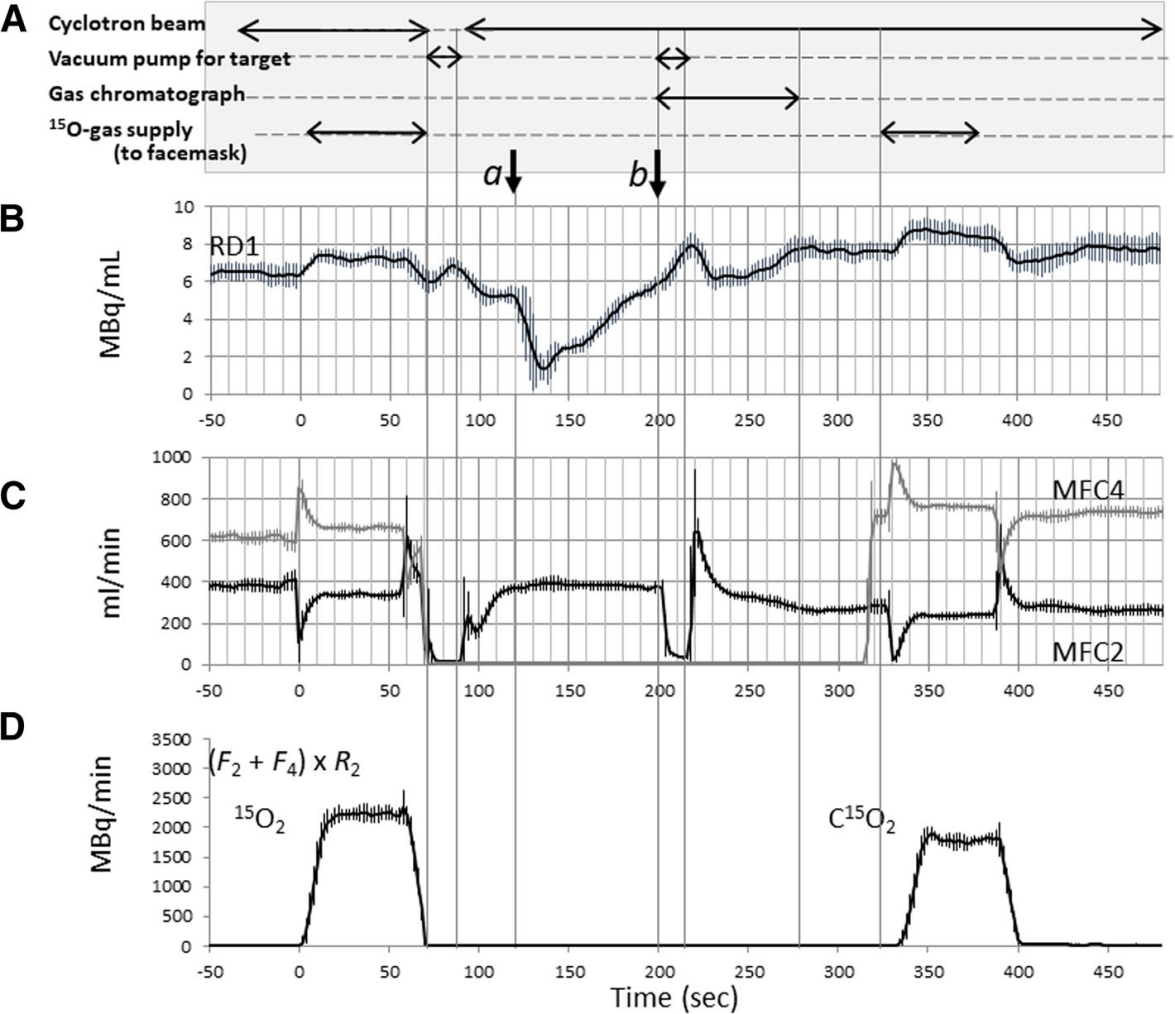
Operation (**a**) and trends of the radioactivity concentrations (**b**), flow rates (**c**), and calculated radioactivity supplied to the face mask (**d**) in the sequential ^15^O_2_–C^15^O_2_ supply protocol. Data are averaged for six independent runs. See text

Figure 3 shows averaged trends of radioactivity concentration of RD1 (Fig. 3b), the flow rates at MFC2 (*F*_2_) and MCF4 (*F*_4_) (Fig. 3c), and the radioactivity supply rate as calculated as (*F*_2_ *+ F*_4_) × *R*_2_ (Fig. 3d) among six experiments, during the sequential ^15^O_2_–C^15^O_2_ supply with given procedures shown in Fig. 3a. The amount of radiopharmaceutical supply was regulated by referring the radioactivity concentration at RD1 and RD2, and also flow rates at MFC2 and MFC4. The total radioactivity supplied to the facemask was 2230 ± 131 MBq and 1796 ± 112 MBq, corresponding to the ^15^O_2_ and C^15^O_2_ inhalation over 1 min, respectively. Supply of ^15^O_2_ to the patient facemask was initiated at *t* = 0 s on the time chart of Fig. 3. The inhalation lasted for 1 min, and then after the gas supply was changed back to the recirculation line, while non-radioactive fresh air continued to be supplied to the facemask to clean the radioactivity. MFC2 and MFC4 were then closed and dropped the flow rates at MFC2 and MFC4 at 70  s. The target gas was then evacuated by a rotary pump for an 18-s period while the target beam was off. When the target was re-flowed with a new gas of N_2_ containing 1% of CO_2_ and the beam was set on. Radioactivity at RD1 was once decreased at approximately 120 s (arrow *a*) because a non-radioactive target gas was carried to the radio-detector RD1, and then gradually increased after 135 s when a new radioactive gas of C^15^O_2_ reached to the line. Flow (MFC2) was stopped (arrow *b*) for approximately 18 s in order to take a sample for the gas chromatography (GC) at 200 s.

Table 1 summarizes the radioactivity yields for 1-min administration of C^15^O, ^15^O_2_, and H_2_^15^O for three of the target gases with different carrier concentrations of O_2_ (0.3%, 0.5%, and 1.0%) in the N_2_ gas. Production yields of ^15^O_2_ and H_2_^15^O were not significantly different among the three O_2_ carrier gas concentrations. However, the C^15^O production was significantly smaller when a 0.3% concentration of O_2_ was selected as compared with 0.5% and 1.0% concentrations. The radioactivity yield was 2976 ± 78 MBq for a C^15^O_2_ production using a target gas containing 1.0% carbon dioxide (CO_2_) in the N_2_ gas with a beam intensity at 40 μA, which was significantly greater than that for an ^15^O_2_ production.

**Table 1:** Radioactivity yields of C^15^O and ^15^O_2_ gases and H_2_^15^O saline for three different oxygen concentrations in nitrogen as the target gas

Oxygen concentration in nitrogen	C^15^O^a^ (MBq)	^15^O_2_^a^ (MBq)	H_2_^15^O^b^ (MBq)
0.3%	1757 ± 146*	2634 ± 145	731 ± 16
0.5%	1974 ± 39	2733 ± 60	735 ± 41
1.0%	2043 ± 69	2749 ± 72	742 ± 14

Note that H_2_^15^O saline was produced by infusing saline over 60 s beginning 2 min after deuteron beam initiation. The sample volume of H_2_^15^O saline was 10 mL

^a^Beam intensity is 40 μA

^b^Beam intensity is 10 μA

Table 2 summarizes the chemical concentrations for each of C^15^O, ^15^O_2_, and C^15^O_2_ productions, in which the target gas was fixed at 0.3% O_2_ in N_2_ for C^15^O and ^15^O_2_ productions, and at 1.0% CO_2_ in N_2_ for a C^15^O_2_ production. Because the target gas was mostly nitrogen gas, the nitrogen concentration is the highest in all three radiopharmaceuticals. It is noteworthy that the CO contamination was the smallest and approximately 0.6% with 0.3% of O_2_ carrier gas in N_2_ target gas. The CO contamination increased when higher concentration of CO is used as a carrier gas. The concentration of CO was approximately 1.0% and 2.0% when the target contained 0.5% and 1.0% of O_2_ as a carrier gas, respectively. No other peaks else than the main peak of the radiopharmaceutical was seen in any of C^15^O, ^15^O_2_, and C^15^O_2_ productions, thus the radiochemical purity was effectively 100% for all compounds.

**Table 2:** Chemical concentration and radiochemical purity

	^15^O-production		
Chemical component	C^15^O (%)	^15^O_2_ (%)	C^15^O_2_ (%)
N_2_	98.77 ± 0.52	99.00 ± 0.43	98.99 ± 0.55
CO	0.60 ± 0.06	0.10 ± 0.07	0.04 ± 0.01
O_2_	0.64 ± 0.56	0.91 ± 0.40	0.99 ± 0.55
CO_2_	0.03 ± 0.03	0.01 ± 0.03	0.82 ± 0.11
Radiochemical purity	100	100	100

**Fig. 4:**
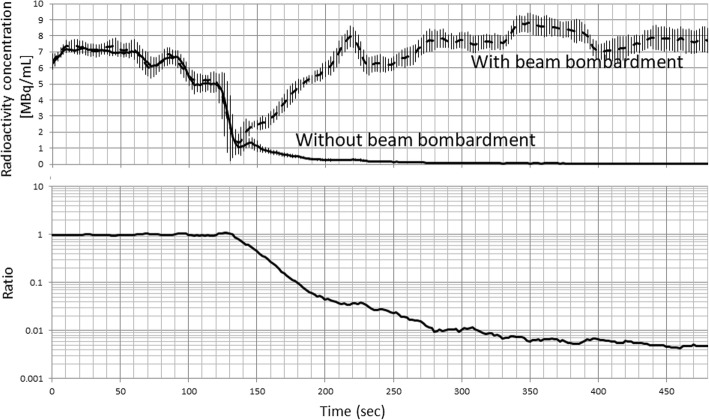
Time-activity curves of RD1 in Fig. 1 with and without cyclotron beam bombardment for the second supply of C^15^O_2_ (upper). Ratio of the two curves indicates the fractional contamination in the C^15^O_2_ radioactivity from the previous production of ^15^O_2_ (lower). Note that the time scale is given after initiation of the ^15^O_2_ supply to the face mask

Figure 4 shows TACs  of the radioactivity detector, RD1 during the sequential ^15^O_2_–C^15^O_2_ supply protocol, which is the same as that shown in Fig. 3, but are plotted for both with and without beam bombardment for the C^15^O_2_ production. A rise in the radioactivity concentration after 135 s was attributed to production of the C^15^O_2_ after the cyclotron beam bombardment. In contrast, the radioactivity continued to be decreased, when the cyclotron beam was not initiated. The ratio of the two radioactivity curves which decreased monotonically determines the amount of contamination when the rapid protocol was employed, and was shown to be 1% at approximately 280 s, and 0.7 ± 0.1% at 330 s which is the time when the C^15^O_2_ inhalation was initiated in the clinical protocol.

**Fig. 5:**
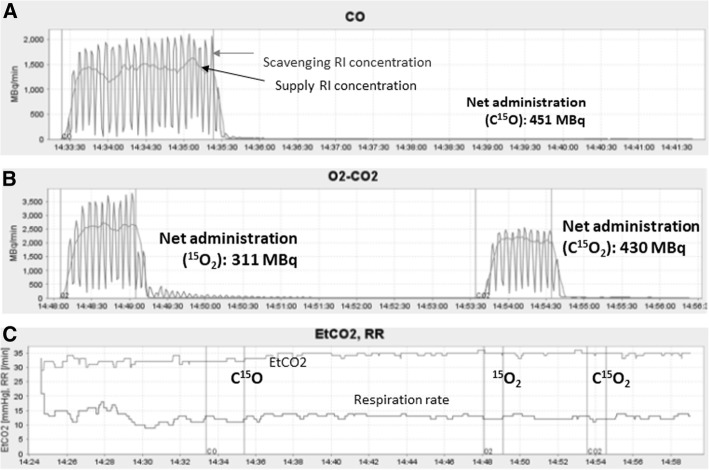
Quantitative assessment of the gaseous radioactivity in the supply and scavenging tubes for the two-hole two-layered facemask in typical C^15^O (**a**) and sequential ^15^O_2_–C^15^O_2_ (**b**) inhalation scans. End-tidal carbon dioxide concentration (EtCO_2_) and the respiration rate (RR) (**c**) demonstrate stable normocapnia during the entire PET examination. Three sets of bands correspond to periods of C^15^O inhalation over 2 min and sequential ^15^O_2_ and C^15^O_2_ inhalation each over 1 min. See text

Figure 5 shows time-activity curves of a pair of detectors obtained in one of example cases, corresponding to the supply and scavenging tubes for a C^15^O inhalation (a) and a sequential ^15^O_2_-C^15^O_2_ inhalation scans (b), respectively. Time-dependent end-tidal carbon dioxide (EtCO_2_) and respiration rate (RR) curves (**c**) are also shown for this particular case. The net administration doses, as calculated as a difference of AUC’s of two curves for this particular case, were 451 MBq, 311 MBq, and 430 MBq for the supply of 2747 MBq, 2451 MBq, and 1883 MBq, corresponding to C^15^O, ^15^O_2_, and C^15^O_2_ inhalation, respectively. Similarly, supply and net administration doses over all 749 cases were 541 ± 149 MBq, 320 ± 103 MBq, and 523 ± 137 MBq for supply of 2574 ± 255 MBq, 2220 ± 766 MBq, and 1763 ± 174 MBq corresponding to C^15^O, ^15^O_2_, and C^15^O_2_ inhalation, respectively (see also Table 3). It should also be noted that EtCO_2_ and respiration rate (RR) are stable during the PET examination period. The mean EtCO_2_ was 37.8 ± 5.9 mmHg and 37.9 ± 6.0 mmHg, and RR 14.0 ± 3.6 and 14.0 ± 3.5 min^−1^, during to the ^15^O_2_ and C^15^O_2_ inhalation periods, respectively. Difference between the two periods was 0.5 ± 1.0 and − 0.6 ± 2.5 mmHg for EtCO_2_ and 0.68 ± 0.75 and 0.03 ± 2.38 min^−1^ for RR, corresponding to healthy volunteers (*n* = 15) and all subjects including patients, respectively.

**Table 3:** Summary of the supply and net administration doses, and the leakage from the face mask system

	C^15^O scan	^15^O_2_ scan	C^15^O_2_ scan
Supplied radioactivity per scan	2574 ± 255 MBq	2220 ± 766 MBq	1763 ± 174 MBq
Net administration per scan	541 ± 149 MBq	320 ± 103 MBq	523 ± 137 MBq
%Leakage	0.166 ± 0.493%	0.181 ± 0.525%	0.148 ± 0.545%
# of scans with no detected leakage	417	395	529
# of scans	749	749	749

**Fig. 6:**
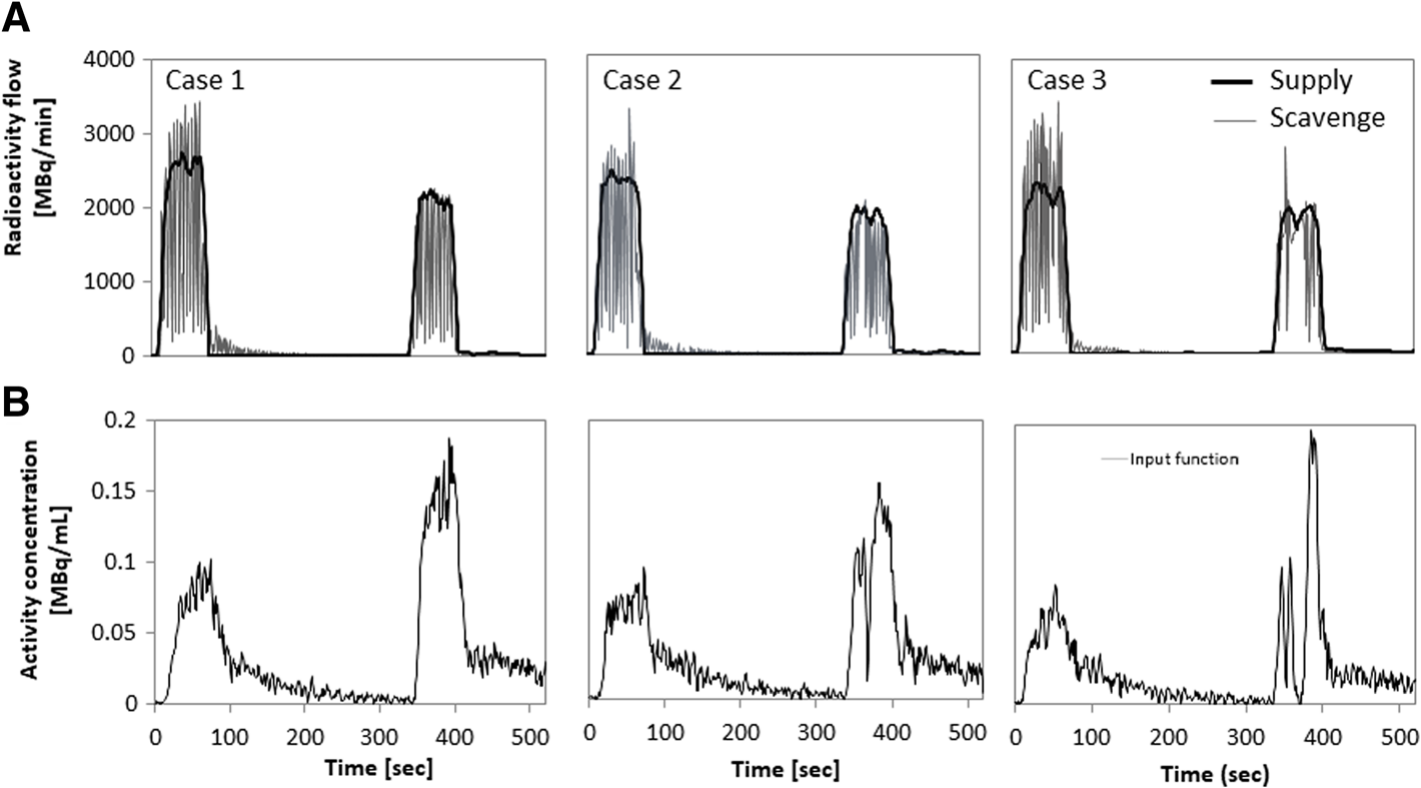
Comparison of the gaseous radioactivity curves in the supply and scavenging tubes (upper) with AIF (lower) assessed with arterial blood sampling in typical three example cases

**Fig. 7:**
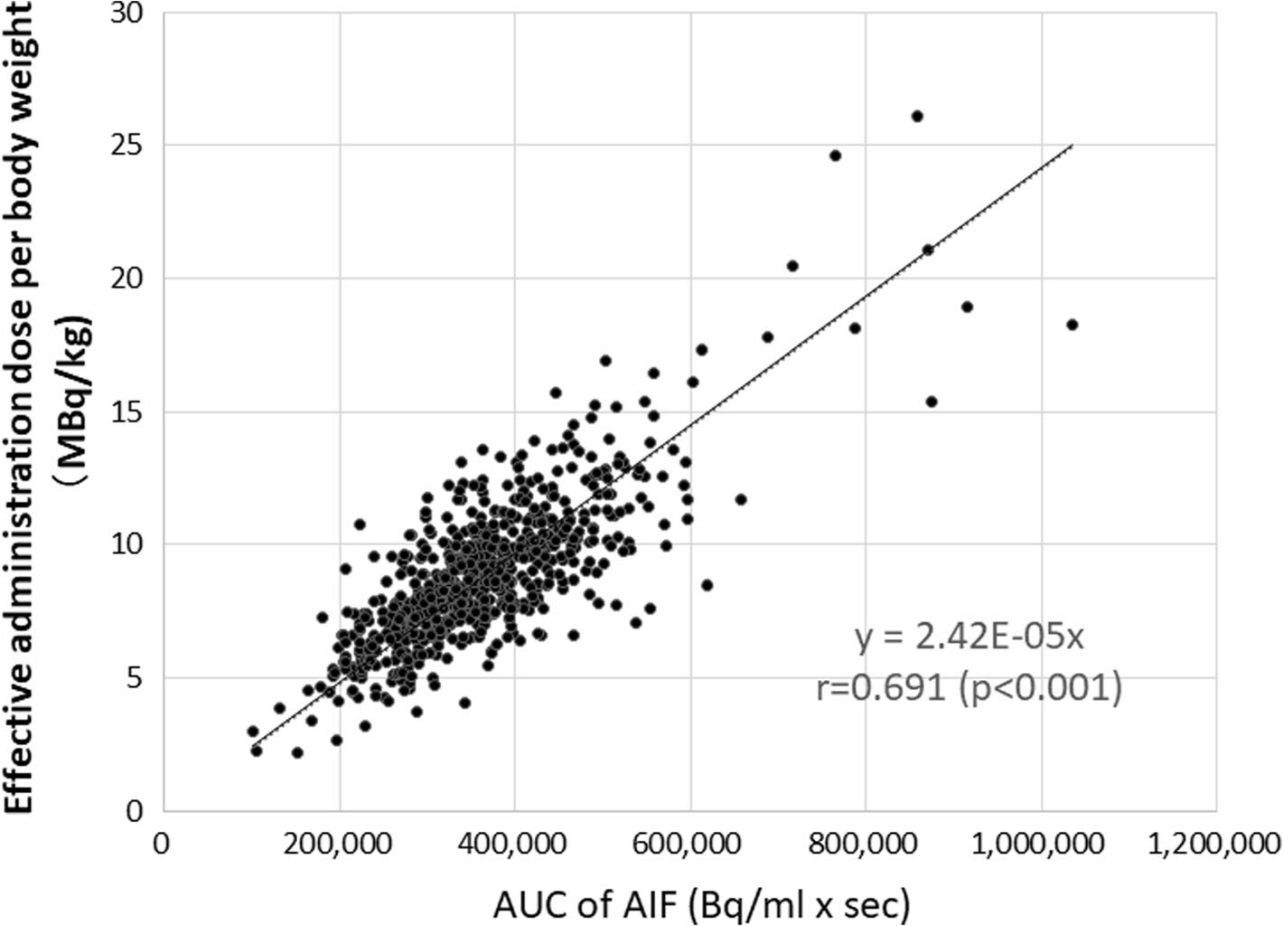
Effective C^15^O_2_ administration dose per body weight as a function of the area-under the curve (AUC) of the calibrated arterial input function (AIF)

Figure 6 shows three sets of example cases of the time-radioactivity curves in the supply and scavenging tubes (a), as compared with AIFs which were assessed directly using the continuous arterial blood sampling (b). Case 1 was from a healthy volunteer who demonstrated a stable inhalation, in which a periodically oscillating radioactivity TAC in the scavenging radioactivity corresponded well to the zigzag shape of AIF. The other two cases demonstrated irregular shapes in the scavenging radioactivity. Pauses of inhalation observed during the C^15^O_2_ inhalation period in cases 2 and 3 were consistent with the drop in AIF. Irregular shapes of AIF attributed to the pause of respiration were observed in 70 and 85 scans during the ^15^O_2_ and C^15^O_2_ inhalation scans out of 749 scans, respectively. Figure 7 showed the estimated net administration dose of C^15^O_2_ divided by the body weight of each patient as a function of AUC of the invasively assessed AIF. The two values were significantly correlated to each other (*p* < 0.001).

**Fig. 8:**
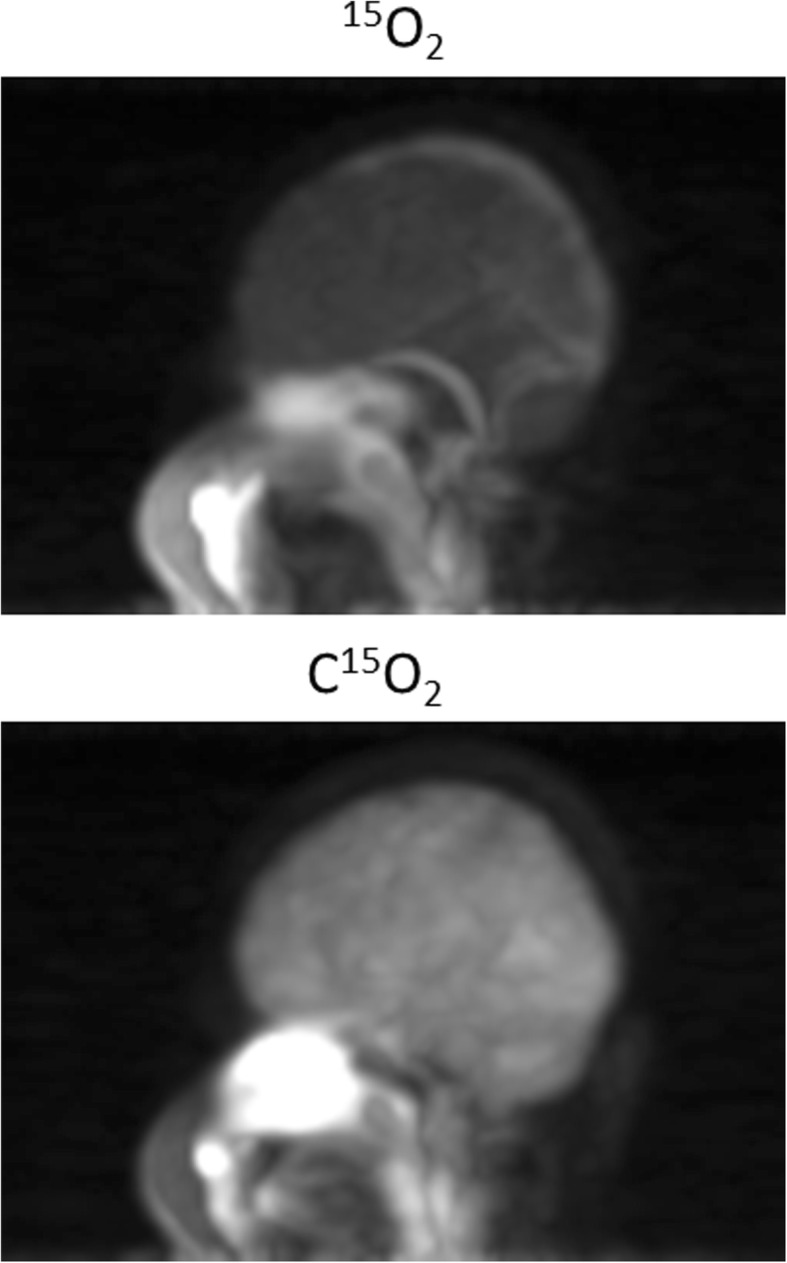
Maximum intensity projection (MIP) images of a patient while breathing ^15^O_2_ and C^15^O_2_. See text

**Fig. 9:**
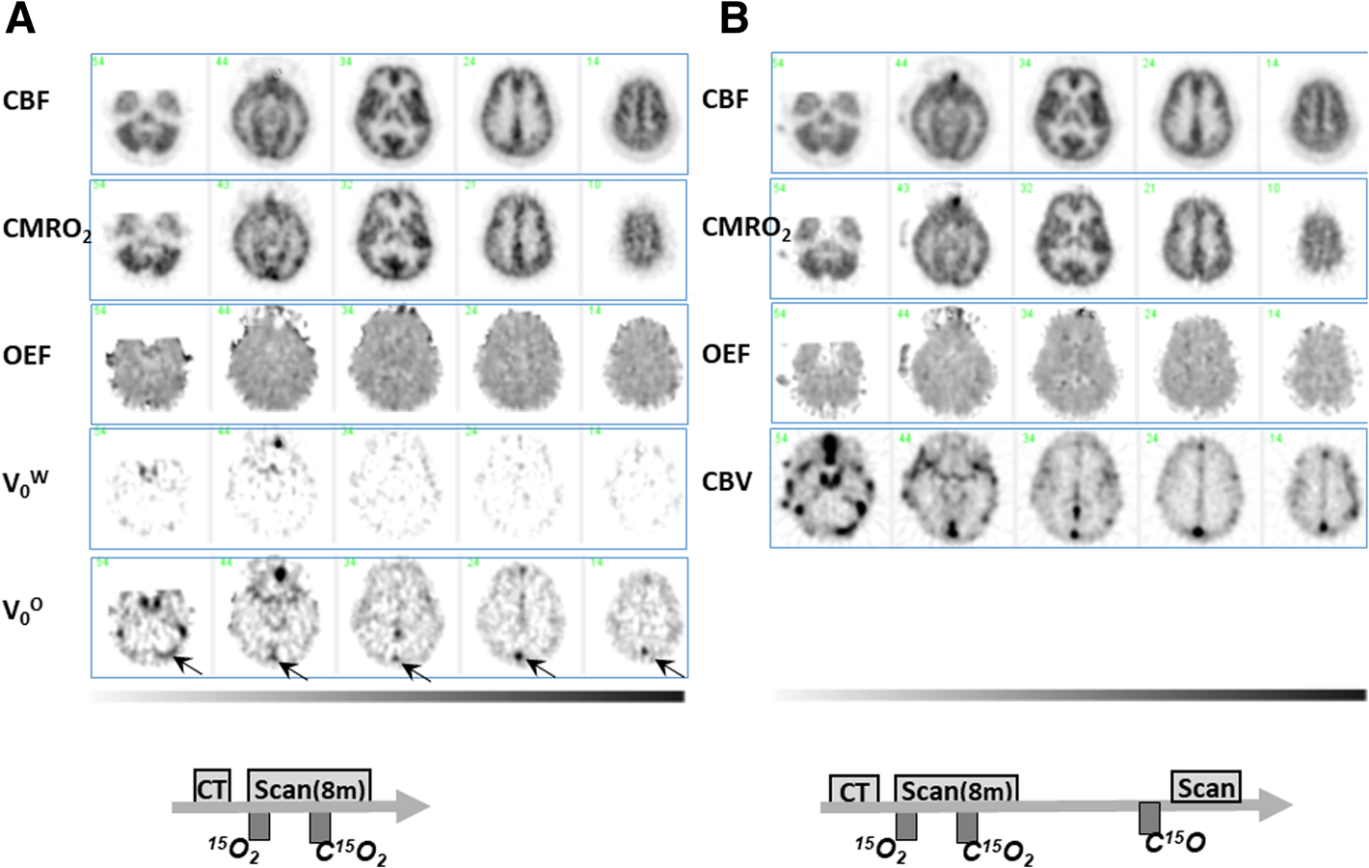
Example functional images of CBF, CMRO_2_, OEF, and vascular volume images of the arterial blood volume for H^15^O (*V*_0_^W^) and that for ^15^O_2_ (*V*_0_^O^), and CBV, obtained from a healthy volunteer of a 22-year-old male of the body weight 68 kg, with the net administration dose 503, 267, and 592  MBq corresponding to C^15^O, ^15^O_2_, and C^15^O_2_ inhalation, respectively. Functional images were calculated according to a modified DBFM [26] (**a**) and a DARG [25] (**b**) methods. Note that the functional images on both A and B were calculated from the same data acquired with the single PET scan during sequential inhalation of ^15^O_2_ and C^15^O_2_, but without C^15^O scan data on (**a**)**.** CBV indicates high counts in the sinus area, while *V*_0_^W^ does not show. *V*_0_^O^ demonstrates significantly high counts. Of note is that image quality was reasonably well in both calculation methods, despite of gaseous radioactivity inhalation protocol

PET images demonstrated that radioactivity in the facemask was well scavenged and was small compared with that in the brain as typically seen in Fig. 8. Identification of the vasculature including the carotid artery was also reasonably well even during the ^15^O_2_ and C^15^O_2_ inhalation periods. Radioactivity in the facemask relative to the brain was higher during inhalation of ^15^O_2_ than that of C^15^O_2_. Larger radioactivity was seen in the paranasal sinus area during C^15^O_2_ inhalation but not during ^15^O_2_ inhalation. The brain uptake relative to the radioactivity in the facemask was higher with C^15^O_2_ than that with ^15^O_2_ inhalation. Figure 9 shows an example of functional images obtained from a healthy volunteer of a 22-year-old male of the body weight 68 kg. The net administration dose on this subject was 503, 267, and 592 MBq corresponding to C^15^O, ^15^O_2_, and C^15^O_2_ inhalation, respectively. Functional images calculated by means of two different mathematical formulations, i.e., DARG [25] and a modified DBFM [26] methods (Fig. 9b), demonstrated similar results, except for some differences including different magnitude of the noise between the two methods, and also between the vascular volume images (*V*_0_^W^ and *V*_0_^O^) and CBV. However, no clear effects of the gaseous radioactivity are visible in both functional images.

**Fig. 10:**
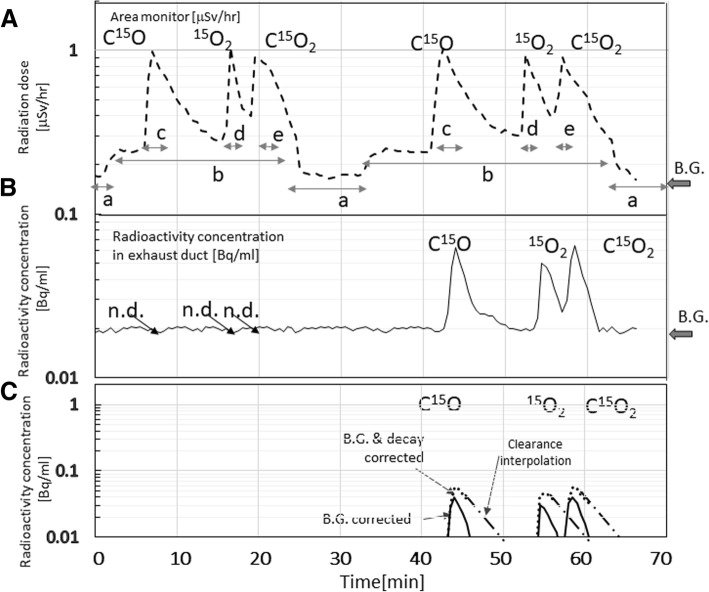
Example of time-activity curves of the gamma-ray monitor detector placed in the PET room (**a**), the radioactivity concentration of the air in the exhaust duct carried from the PET scanner room (**b**), and background- and decay-corrected radioactivity curves in the exhaust duct (**c**). Data were observed during a series of PET examinations involving C^15^O, ^15^O_2_, and C^15^O_2_ inhalation on each of two patients. Characters “a” and “b” corresponded to a period when the cyclotron was accelerating a deuteron beam to a target, in which the radioactive gas was not circulated to the PET room during period “a,” but it was circulated to the PET room during period “b.” The three other characters “c,” “d,” and “e” corresponded to the periods of supplying C^15^O, ^15^O_2_, and C^15^O_2_ gases to the patient’s facemask, respectively. Leakage of gaseous radioactivity was not detected (n.d.) during the first examination, but was significant in the second examination as shown in (**b**)

Figure 10 showed time-dependent radioactivity curves, measured using a gamma detector in the PET scanner room (a), and the gaseous radioactivity concentration in the exhaust duct (b), observed during two sequential PET examinations involving a series of C^15^O, ^15^O_2_, and C^15^O_2_ inhalation on each of two patients. The area radioactivity in the PET room slightly increased during the period when the radioactive gas was circulated to the PET room during the periods indicated by “b” in (a). The radioactivity in the PET room increased immediately after inhalation of each of C^15^O, ^15^O_2_, and C^15^O_2_ gases, indicated by “c,” “d,” and “e,” respectively. Sharper peak was observed after ^15^O_2_ inhalation as compared with after C^15^O and C^15^O_2_ inhalation. Gaseous radioactivity was observed in the exhaust duct during the second examination, which indicated the leakage from the facemask when gaseous radiopharmaceuticals were supplied (b). The radioactivity curves in the exhaust duct corrected for background (b), and decay of ^15^O were plotted in (c). The %leakages were summarized in Table 3 for each of C^15^O, ^15^O_2_, and C^15^O_2_ inhalation scans. Leakage was not detected in more than a half of scans, namely 417, 395, and 529 scans with C^15^O, ^15^O_2_, and C^15^O_2_ inhalations, respectively. Three examinations demonstrated that the leakage reached 1%, but the average leakage was 0.17 ± 0.65% when corrected for the radioactivity decay.

## Discussion

The present study demonstrated that the novel radiosynthesis system was able to produce and supply a sufficient amount of a series of ^15^O-labeled radiopharmaceuticals of C^15^O, ^15^O_2_, and C^15^O_2_ by combining it with the ^15^O-dedicated small cyclotron of C3D when a deuteron beam was accelerated at a beam current of 40 μA (Tables 1 and 3). The radiochemical purity was sufficiently high with acceptable chemical contaminations (Table 2). The sequencer-controlling radiosynthesis and the inhalation controller allowed a reliable operation and fairly good reproducibility in the production and inhalation of a series of radiopharmaceuticals. The radiochemical contamination was shown to be negligibly small even when the radiopharmaceuticals were supplied at 4.5-min intervals (Fig. 4). These features allowed the recently developed rapid method of Kudomi et al. [26] applicable to PET imaging under the clinical environment, as has been demonstrated in more than 2500 examinations in our facility since December, 2012. Automatic evacuation of the target gas that was carried immediately after the end of production of a previous radiopharmaceutical was effective to reduce contamination from the previous supply in the second production despite of the short interval. Automatic record of radioactivity detectors, electronic valves, flow meters, the pressure monitoring sensors, together with other monitoring devices needed in PET examinations were aimed to ensure the quality of ^15^O-labeled radiopharmaceuticals in routine clinical scans. This feature would assist the quality of radiopharmaceuticals as a part of the good manufacturing practice (GMP), and the standardization of PET examination.

An important outcome of the inhalation/scavenging unit implemented in this system was the ability to remove radioactivity from the area inside the FOV of PET imaging by effectively scavenging the non-inhaled radioactive gases from the outer layer of the two-hole two-layered face mask (Fig. 2), while a sufficient amount of radiopharmaceuticals were confined in a patient nose/mouth area as demonstrated in Figs. 8 and 9. It was shown earlier that the present inhalation/scavenging unit was effective to suppress the random rates which might be caused by a relatively strong gaseous radioactivity, contributing to reduced dead-time, and thus improved quantitative accuracy with minimal enhancement of image noise, as shown in our earlier study [22]. It is also important to note that the improved accuracy of PET images during the gaseous ^15^O-labeled radiopharmaceuticals may enable the determination of AIF from radioactivity concentration curves in the carotid arterial region. This may further provide opportunity of a non-invasive quantitation of functional parametric images without the arterial blood sampling [23]. Systematic study should be carried out to evaluate the adequacy of the non-invasive quantitation in ^15^O-oxygen PET scans.

The scavenged gaseous radioactivity was compressed and stored in the decay tank (tank 1 in Fig. 1), until it decayed sufficiently prior to releasing it to the atmosphere. This was aimed to prevent from the radiation of not only staff working in the PET facility but also a public living outside of the facility. It is also important to note that use of the present inhalation/scavenging unit allowed for a stable respiration or normocapnia during an entire PET examination period, as typically seen Fig. 5c. It has been reported that only a change of 1.0 mmHg in PaCO_2_ causes a change by 6% in CBF [31], but the present system allowed only a small change in EtCO_2_, which is an indirect marker of PaCO_2_, between ^15^O_2_ and C^15^O_2_ inhalation periods, e.g., 0.5 ± 1.0 and − 0.6 ± 2.5 mmHg, corresponding to healthy and patient populations, respectively. The variations in EtCO_2_ of ± 1.0 mmHg between the ^15^O_2_ and C^15^O_2_ inhalation periods in healthy controls were practically the same. In the entire subjects including the patient population, the variation was larger and reached to ± 2.5 mmHg, which was attributed to the unstable breathing. As typically shown in Fig. 6, more than 10% of patients displayed AIFs with a zigzag shape, which was due to unstable breathing. The net administration curves and AIFs were less reproducible among subjects in patient populations, as also seen in EtCO_2_ curves which were unstable in patients. The transient change in PaCO_2_ during the PET examination would still be in an acceptable range in order to reliably quantify CBF and CMRO_2_ in clinical settings. Continuous supply of a fresh air to the two-hole two-layered facemask assisted easy breathing despite of prevention from the leakage of radiopharmaceuticals, as seen in Fig. 5c.

It can be seen from Fig. 7 that the net administration dose can be estimated as the difference in the radioactivity between the inlet and outlet tubes for the C^15^O_2_ inhalation scan. Transient changes in inhaling the gaseous radiopharmaceuticals suggested that population-based AIFs were limited in terms of providing individual AIFs. It should also be noted that the net administration might be overestimated when ^15^O_2_ is inhaled, because ^15^O_2_ stays longer as a gaseous form in the lung cavity than C^15^O_2_ and C^15^O before transported to the blood circulation. This could be modeled, but it is beyond of the scope of the present study. Further studies need to be carried out.

A larger radioactivity in the nasal cavity during C^15^O_2_ inhalation is attributed to the solubility of carbon dioxide compared to that of ^15^O_2_, though this did not have a significant contribution to degrade the image accuracy or image quality. A higher accumulation of C^15^O_2_ into the brain than that of ^15^O_2_ is attributed to the higher extraction of gaseous radioactivity in the lung to the blood circulation, and also to a higher first-pass extraction rate of H_2_^15^O than that of ^15^O_2_ in the brain. Larger radioactivity inside the facemask during the ^15^O_2_ inhalation than that during C^15^O_2_ inhalation could be explained by a smaller extraction rate of ^15^O_2_ in the lung than that of C^15^O_2_. The gamma detector curves in the PET room showing a sharper peak or faster clearance after the ^15^O_2_ inhalation than that of C^15^O_2_ and that of C^15^O was attributed to the smaller fraction of inhaled ^15^O_2_ were absorbed in the lung being transferred to the blood circulation. In other words, more amount of ^15^O_2_ was scavenged from the facemask, than other radiopharmaceuticals.

It is worth noting that the chemical contamination of non-radioactive carbon-monoxide in the C^15^O radiopharmaceutical varies dependent of the carrier gas concentration of oxygen in the target gas. This is because the total amount of carbon-monoxide becomes double of the oxygen content as two molecular units of CO are produced from one O_2_ molecule by combining it with two carbons in the charcoal column. The use of 0.3% of cold oxygen in nitrogen gas should therefore be encouraged to minimize the toxicity of the carbon-monoxide contamination instead of 0.5% or 1.0% O_2_, which produces 1.0% or 2.0% of CO, respectively. This study demonstrated that the radioactivity yields were not significantly different in the ^15^O_2_ and H_2_^15^O production, although the production of C^15^O was reduced, but only approximately 10%, which should not cause significant problems in practical examinations.

The present study demonstrated that the average leakage was 0.17 ± 0.65% from the facemask as estimated in this study including corrections for backgrounds and the decay (Fig. 10). Due to the regulation required by the radiation safety authority in this country, the room air had to be evacuated at approximately 2000 m^3^ per hour to the exhaust ducts, which corresponded to 17 times of exchanging the entire air in the PET room. Such strong evacuation may not be needed by using the present two-hole two-layered facemask with active scavenging. Use of the decay tank, tank2 in Fig. 1**,** should be encouraged in order to minimize the leakage to the room in the facility and also to the public. The International Commission on Radiological Protection (ICRP) does not define the upper limit of radioactivity nor a radioactivity concentration at the exhaust duct for short half-life radio-nuclei in gaseous form. This is because the public safety may be guaranteed by stopping the room exhaust if significant leakage occurs. In such a situation, the workers in the facility would have to leave from the area to prevent from a high risk of radiation. However, this is not practically adequate, because clinical examinations are carried out on patients inside the facility. To reduce the radioactivity released from the exhaust duct to the public, the use of decay tank was shown to be effective, in which use of the facemask that prevents from the leakage to the room was essential. The present inhalation/scavenging unit which reduced the leakage to the order of 0.1% was essential when utilizing the gaseous ^15^O-oxygen radiopharmaceuticals in clinical PET examinations.

## Conclusions

The present study demonstrated that the recently developed system for automated production and inhalation of a series of ^15^O-labeled gaseous radiopharmaceuticals produced enough amount of C^15^O, ^15^O_2_, and C^15^O_2_ at a reasonably short interval with sufficiently high radiochemical purity as required for clinical use. It is also important to note that logistical complexity in terms of producing and inhaling three sets of ^15^O-Oxygen-labeled radiopharmaceuticals has been considerably improved. This is particularly essential when a recently proposed rapid protocol of Kudomi et al. [26] is utilized under clinical environment. Functional parametric imaging of CBF, CMRO_2_, and OEF can therefore be obtained from a short scan period of 8 min, with considerably reduced work load of clinical staff as compared with previous PET examination systems. Effective scavenge allow for accurate quantitation using a commercial PET scanner in 3D mode. Minimal leakage from the facemask should contribute to reduction of unnecessary radiation for workers and for public.

## Abbreviations

AIF: Arterial input function
AUC: Area under the curve
CBF: Cerebral blood flow
CBV: Cerebral blood volume
CMRO_2_: Cerebral metabolic rate of oxygen
EtCO_2_: End-tidal carbon-dioxide
MFC: Mass flow controller
MIP: Maximum intensity projection
MS: Molecular Sieve 5A
OEF: Oxygen extraction fraction
PPQ: Pora-Plot Q
RD: Radio-detector
RR: Respiration rate
RWG: Radio water generator
TAC: Time-activity curve
